# Study on the Biodegradation Process of D-Mannose Glycopolymers in Liquid Media and Soil

**DOI:** 10.3390/polym15153194

**Published:** 2023-07-27

**Authors:** Ana-Maria Pană, Valentin Ordodi, Vasile Gherman, Paula Sfîrloagă, Gabriela-Alina Dumitrel

**Affiliations:** 1 Faculty of Industrial Chemistry and Environmental Engineering, Politehnica University Timişoara, 2 Victoriei Square, 300006 Timişoara, Romania; anamaria.pana@upt.ro (A.-M.P.); valentin.ordodi@upt.ro (V.O.); vasile.gherman@upt.ro (V.G.); 2 The Institute of Research for Condensed Matter, 1 P. Andronescu Street, 300224 Timişoara, Romania; paulasfirloaga@gmail.com

**Keywords:** biodegradable polymers, *P. mirabilis*, soil biodegradation, glycopolymers

## Abstract

Polymers derived from natural raw materials have become of great interest due to their increased biodegradable features and possible biocompatibility. Our group has successfully synthesized and characterized polymers derived from D-mannose oligomer (M), 2-hydroxy propyl acrylate (HPA), and methacrylate (HPMA) in different weight ratios. Their biodegradation was studied in liquid media with pure *Proteus mirabilis* inoculum for the samples with the most sugar residue, and the results show that the methacrylate derivative M_HPMA1 lost about 50% of its weight during incubation. SEM/EDX techniques were employed to display the modifications of the samples during the biodegradation process. The glycopolymers were buried in garden soil, and the experiment proved that more than 40% of the weight of the M_HPA1 sample was lost during biodegradation, while the other samples encountered an average of about 32% weight loss. The biodegradation profile was fitted against linear and polynomial mathematical models, which enabled an estimate of about a year for the total degradation of the D-mannose glycopolymers sample in soil.

## 1. Introduction

Along with industrial development and technological advancements, the need for versatile materials has increased dramatically [1]. Plastic materials have emerged as light-weight, electrically, and thermally insulating, good-strength alternatives with applications in various fields like automotive, construction, agriculture [2], food, medicine, etc., and have become irreplaceable in everyday life [3,4]. The main flaw of plastic materials is that their life span is very small compared to the time required for their degradation in environmental conditions; therefore, their footprint is recognizable and traceable for decades [5,6]. Moreover, in order to improve their versatility, in the manufacturing process certain amounts of other chemicals are added (i.e., catalysts, dyes, plasticizers, flame retardants, etc.), which can also lead to contamination of the environment with hazardous compounds [7].

Certain statistics have shown that more than 70% of the plastic collected in the EU will be mostly incinerated or landfilled, while the rest will eventually be successfully recycled and reintroduced to the ever-increasing consumer market [8,9]. The growing production rate of plastic production and therefore release has become a threat to ecosystems and a high concern regarding the ability to mitigate their long-term effects on the environment [10,11].

In light of the above, certain measures have been discussed by international governments in order to address the problem of increasing quantities of plastic waste [12]. The presence of biodegradable plastic materials on the market has drastically increased as a result of certain policies and regulations in the EU and abroad [13].

Biodegradability is defined as the ability of a material to degrade into simple matter that can be metabolized by microorganisms as a carbon source under the required conditions [14]. The mechanism of plastic materials’ biodegradation is yet to be established [15]. Research in the field has concluded so far that biodegradation occurs in three different steps: (i) development of a biofilm onto the polymeric surface; (ii) microorganisms’ action towards the polymeric chain and its scission due to enzymatic mechanisms; (iii) metabolization of polymeric residues as carbon sources [16].

The biodegradation of plastic materials has been a matter of increased interest in the past decades, as it should improve the environmental impact of these materials [17]. The mechanism of degradation is linked to the metabolism displayed by the microorganisms involved, whether aerobic (in the presence of oxygen) or anaerobic (without oxygen), along with the development of colonies able to degrade and use polymeric chains as carbon sources and the subsequent release of by-products (i.e., CO_2_, CH_4_, respectively, water, biomass, humic materials, etc.) [18,19]. Recently, research in the degradation of plastic materials has been carried out using certain larvae or worms that can contribute to the scission of polymeric chains and make them available for subsequent alteration in the presence of bacteria and/or fungi [20]. The key to the biodegradation process is the availability of certain bonds between monomers (esteric, amidic, etc.) to enzyme attack, produced by certain microorganisms, which would produce scission into the long-ordinated polymer chain. Then, the resulting fragments could be of use as carbon sources for certain microorganisms, especially if naturally occurring molecules (i.e., saccharides, amino acids, etc.) are recognized as part of chains [21].

For more than two decades, groups of researchers all over the world have been involved in the synthesis of new polymers with enhanced biodegradable features [22], derived from natural raw materials, and able to display good thermal and mechanical strength [23,24]. Bioplastics are either biobased or biodegradable polymers or display both properties [25]. Although bioplastics account now for less than one percent of the total annual production of plastic materials, the market is envisioned to expand to about 7.5 million tons in 2026 [26].

Sugar-based polymers, generally referred to as glycopolymers, are a promising replacement for hydrocarbon-derived classical plastic materials [27,28], along with other natural building blocks (i.e., proteins, lipids) [29]. The synthesis of glycopolymers is based on chemical alterations of the sugar ring with the purpose of attaching C=C double bonds susceptible to polymerization in different conditions. Scientists have approached different sugar moieties, ranging from monosaccharides (i.e., glucose, mannose, ribose, galactose, etc.), disaccharides (i.e., sucrose, trehalose, etc.), or polysaccharides with various molecular weights (i.e., chitosan, starch, cellulose, etc.). The obtained materials proved to have good properties, which recommended them for different applications in fields like medicine, as tissue replacement or drug delivery candidates [30], agriculture, food packaging, etc. [31,32,33].

Our group has successfully synthesized different sugar-based polymers, which have proved to behave as prolific plastic materials from a thermal and mechanical point of view while displaying susceptibility to common microorganisms in order to degrade in liquid media [34,35,36]. The work herein studies the biodegradation properties of cross-linked glycopolymers based on a D-mannose oligomer (M) copolymerized with hydroxypropyl acrylate (HPA) or hydroxypropyl methacrylate (HPMA) in different weight ratios. The polymers containing the most sugar skeleton, M_HPMA1 and M_HPA1 (weight ratios M: HPMA = 1:1 and M: HPA = 1:1), were tested for biodegradation availability in liquid medium using *P. mirabilis* pure culture. The whole series of glycopolymers derived from M and HPA and HPMA, respectively, in weight ratios ranging from 1:1, 1:2, 1:3, and 1:4, were tested for biodegradation by burial in garden soil for more than 100 days at constant temperature (25 °C) and moist.

## 2. Materials and Methods

The sugar polymers were synthesized and characterized in our laboratory (Organic Technology Laboratory from Politehnica University Timişoara, Romania), and their features are presented in previously published papers [35,36,37].

### 2.1. Biodegradation Using a Pure Culture of P. mirabilis

The biodegradability of the polymeric samples was investigated in liquid media in vitro using pure cultures of *Proteus mirabilis* (ATCC 25933). The polymeric samples (10 to 20 mg) are weighed, sterilized against the culture medium (120 °C, 20 min), and then submitted to biodegradation for 14 days at 37 °C in a liquid medium inoculated with *P. mirabilis* (2 mL pure inoculum). After the given time, the samples were removed from the medium, washed several times with distilled water, sterilized by immersing in 96% (w.) ethanol, rinsed with distilled water, and air dried until a constant mass. The culture medium for *P. mirabilis* is a glycosylated broth with the following composition: meat extract (3 g/L); peptone (10 g/L); NaCl (5 g/L); glucose (100 g/L); and distilled water (up to 1000 mL). The final pH of the broth is 7.4 ÷ 7.6. All assessments were performed in triplicate. The biodegradation of the samples was assessed by weight loss calculation, and their aspects were analyzed by SEM/EDX and microscopy (FEI Company, Eindhoven, The Netherlands).

### 2.2. Biodegradation in Soil

The glycopolymer samples submitted to biodegradation by burial in soil were weighed (with an initial weight of about 100 mg), then buried at about 20 cm depth in soil for about 105 days. The samples were taken out of the soil at the given time (t_1_ = 30 days, t_2_ = 75 days, t_3_ = 105 days), washed with water, dried, and weighed. The humidity of the soil was about 45%, the granulometry profile displayed particles ranging predominantly between 0.2 ÷ 2 mm and 0.02 ÷ 0.2 mm, and the measured pH was 6.7 ± 0.2 [38,39]. The biodegradation was performed at room temperature (25 ± 1 °C), and the biodegradation susceptibility was assessed by weight loss. All measurements were done in triplicate.

### 2.3. SEM/EDX Analysis

The glycopolymers submitted to biodegradation using pure cultures were investigated using scanning electron microscopy coupled with energy-dispersive X-ray spectroscopy (Inspect S + EDAX Genesis XM 2i, FEI Company, Eindhoven, The Netherlands). After thorough washing with distilled water, glycopolymer samples (10÷20 mg) were mounted on carbon double adhesive stubs. The samples were then sprayed with compressed air to avoid contamination of the electromagnetic column and inserted into the microscope chamber, where they were analyzed in high-vacuum mode.

### 2.4. Mathematical Modeling

Matlab software packages (version: 9.11.0.2022996 (R2021b) Update 4) were employed for regression modeling of data obtained from the biodegradation of glycopolymer samples in soil.

In all cases discussed, it is acknowledged that weight loss (%) is calculated as:

$$w\left(\%\right)=\frac{w_{0}-w_{t}}{w_{0}}\cdot \;100$$

where:

*w* (%)—weight loss;

*w*_0_—sample weight at the beginning of the experiment, g;

*w_t_*—sample weight at a given time, g;

*t*—time, days.

## 3. Results and Discussions

Polymers based on renewable resources, like sugars, have proven to be good biocompatible and biodegradable candidates for common synthetic plastic materials. The glycopolymers derived from D-mannose oligomers isolated in our laboratory and 2-hydroxypropyl acrylate and methacrylate have been tested before for biodegradation susceptibility in the presence of pure strains of microorganisms like *Trichoderma reesei* (a fungus with enhanced activity of the cellulase enzyme) and *Zymomonas mobilis* (a bacteria involved in bioethanol production) [35]. The biodegradation process ran on good terms, so it was decided to try testing a heterotrophic microorganism in order to see if the hydrocarbon backbone could be identified and used as a carbon source as well. Heterotrophs are a group of microorganisms (bacteria, yeasts, molds, etc.) that generally metabolize organic carbon as a nutrient. The liquid environment for the development of the *P. mirabilis colony* was adjusted, as the protein glycosylated broth is required for the early stage of heterotroph adjustment [40]. *P. mirabilis* is a gram-negative rod-shaped bacterium originally classified in the *Enterobacteriaceae* family, but recently (2016) it has been attributed to the *Morganellaceae* family [41].

*Proteus mirabilis* is part of the *Gammaproteobacteria,* which naturally occurs in the gut of mammals and humans and is often linked to certain dysfunctions in the digestive tract (i.e., diarrhea, Cohn disease), urinary tract, wounds, etc. It has the ability to develop in anaerobic conditions, and it is found in soil and water (especially if close to animal farms). The bacteria were named *Proteus* after a character from Homer’s Odyssey who managed to escape by changing its form; *mirabilis* is derived from Latin with the meaning “wonderful”. The metabolic ease of adjustment skills turns *P. mirabilis* into a resilient species that can be traced mainly to sewage, polluted water, and soil [42]. Since most plastic materials end up in polluted areas like sewage water, landfills, etc., where a significant microorganism flora rich in fecal pathogens is detected, the biodegradation experiments focused on the study of glycopolymer samples’ behavior in these conditions.

**Figure 1:**
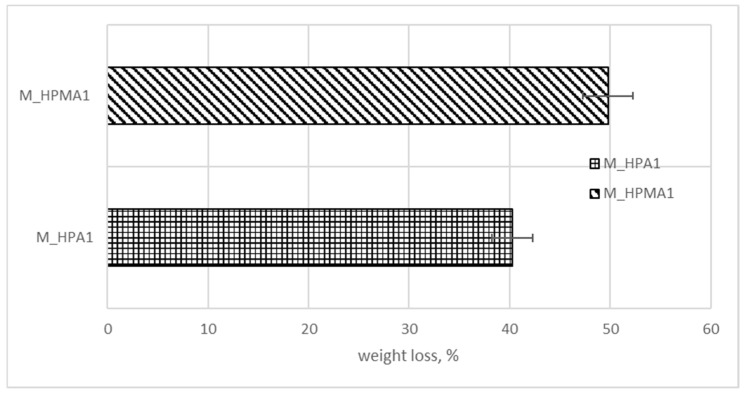
Weight loss of glycopolymer samples submitted to biodegradation in liquid media containing pure *P. mirabilis* strain.

Figure 1 presents the weight loss of the glycopolymer samples based on the D-mannose oligomer (M) and HPA/HPMA in a weight ratio of 1:1 after two weeks of incubation without shaking at about 37 °C in closed test tubes. The polymer containing methacrylate seemed more susceptible to *P. mirabilis* attack, maybe due to larger spaces between cross-linked polymeric chains, a fact also explained by greater water intake capacity. M_HPMA1 displayed a water intake capacity of about 14.4%, while M_HPA1 had a value of about 12.3% at room temperature in distilled water. It must be remarked that in the presence of other bacteria, in past biodegradability assays, it was the acrylate derivative that lost the greatest weight percent [35].

**Figure 2:**
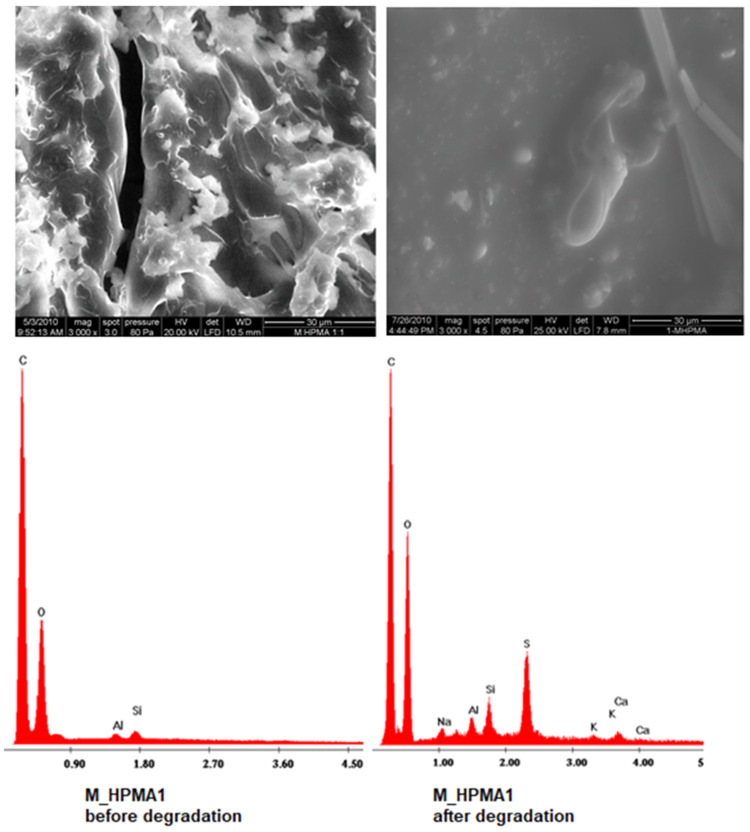
SEM images and EDX spectra of M_HPMA1 glycopolymer before and after biodegradation in liquid media containing a pure *P. mirabilis* strain.

The cross-linked structures of the polymers present hydrophilic pendant units, which enable water intake and thus permit the insertion of microorganisms inside the polymeric matrix. The SEM image presented in Figure 2 exhibits the changes that occurred on the surface of the polymer sample submitted to biodegradation. The SEM picture taken after biodegradation can identify specific traces of *P. mirabilis* colonies on the surface of the polymer, thus explaining the compatibility of this strain to be able to degrade the sugar backbone.

The EDX spectra taken before and after the incubation with a *P. mirabilis* strain have shown that a significant increase in sulfur is detected while the heterotrophic microorganisms have developed in the test tube, along with a meaningful decrease in the weight ratio C:O (from about 3:1 to 2.2:1), due to the fact that the colony has been using the polymeric material as a carbon source for proliferation (Figure 2). The sulfur presence on the EDX diagram is due to the hydrogen sulfide (H_2_S) gas that *P. mirabilis* is generally excreting during metabolism. This common feature can be useful for recognizing the strain [42].

**Figure 3:**
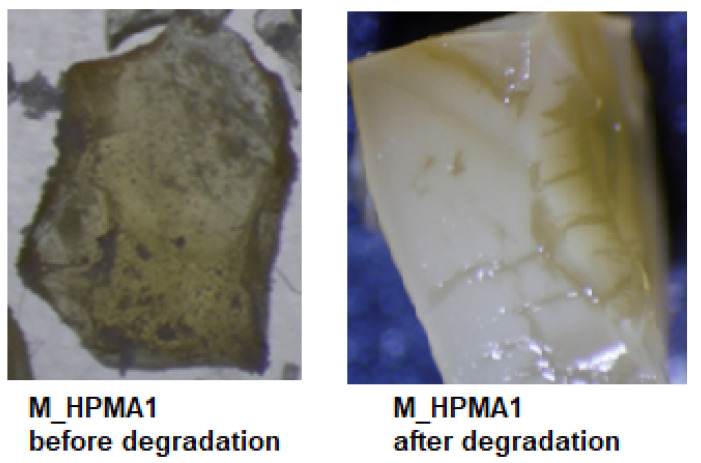
Aspect of M_HPMA1 sample before and after degradation in liquid media in the presence of *P. mirabilis* strain.

As a result of microorganism action, the glycopolymer samples have indicated drastic changes in appearance, displaying frailty, a lack of transparency, and spongy-like features (Figure 3). Also, the aggregation of the microorganism in the form of a sticky, transparent film covering the polymeric sample can be detected, a particularity specific to *P. mirabilis* colonies [42].

When it comes to disposing of plastic materials after use, the environmental laws imply green processes like recycling, energy recovery, and reuse, while more than 30% actually end up in landfills. Our group has tried to mimic the fate of glycopolymers after their life span by studying their behavior after burial in soil [18]. The glycopolymer samples submitted to this test were ground to dimensions of about 100 mg.

A typical garden soil was used, and the glycopolymer samples derived from the D-mannose oligomer and 2-hydroxypropyl acrylate and methacrylate in different weight ratios (1:1, 1:2, 1:3, and 1:4) were employed. The humidity of the soil was maintained at a constant level, and the temperature was about 25 °C.

Table 1 presents the weight losses of the glycopolymer samples analyzed after 30, 75, and 105 days of being inserted into the soil with the specifications presented above. The weights of the samples were reduced (about 100 mg) due to the fact that most waste processors usually grind the wastes and cover them with successive layers of soil to speed up their biological inactivation in landfills [43].

After 30 days, the results indicated that more than 10% of the weight had been lost by burial. The exception is registered for the M_HPA1 sample, which encountered about 18% mass loss, and the M_HPMA4 sample, with a little more than 9%. These exceptions can explain that the sugar content is directly responsible for biodegradation susceptibility in soil. After another 45 days (a total of 75 days), the weight loss tendency is maintained as the values are situated between 12 and 28%, with the glycopolymers containing the most sugar moiety displaying the best results.

The polymers containing the most significant sugar oligomer content displayed the highest biodegradation susceptibility after 105 days of burial, as the M_HPA1 sample lost almost 42% of its mass during the process, while its homologue, M_HPA2, lost almost 38%. The weight loss trend is decreasing as the acrylate moiety content increases, but nonetheless, during the 105 days of observation, almost 27% of the total weight was lost. The experiment was discontinued after 105 days because, for longer periods of time, the samples became very frail and were practically impossible to dig out in full.

The methacrylate derivatives with the same oligomer showed patterns of biodegradation similar to acrylate derivatives, but their weight loss is more modest, with around 37% of their mass being altered by M_HPMA1 (Table 1). The glycopolymer sample containing the D-mannose oligomer and the methacrylate in the greatest percent (M_HPMA4) showed degradation of about 25% of its mass after 1005 days of burial in soil, while the homologue containing the most sugar content reached about 37% of its weight.

Modeling and simulation of biotechnological processes has become a challenge for the research community as more technologies insert biological steps into production as part of a trend to turn more towards a circular economy [44]. Understanding these processes and the interdependency of factors, especially the biological rate-determining step, is the key to better production results [45].

Biodegradation of glycopolymer samples by soil burial was quantified by weight loss calculus as a definitive measure of the extent of the process. The data presented in Table 1 were analyzed, and it was decided to apply simple mathematical equations (i.e., linear and polynomial) in order to obtain information regarding the kinetics of the process.

**Figure 4:**
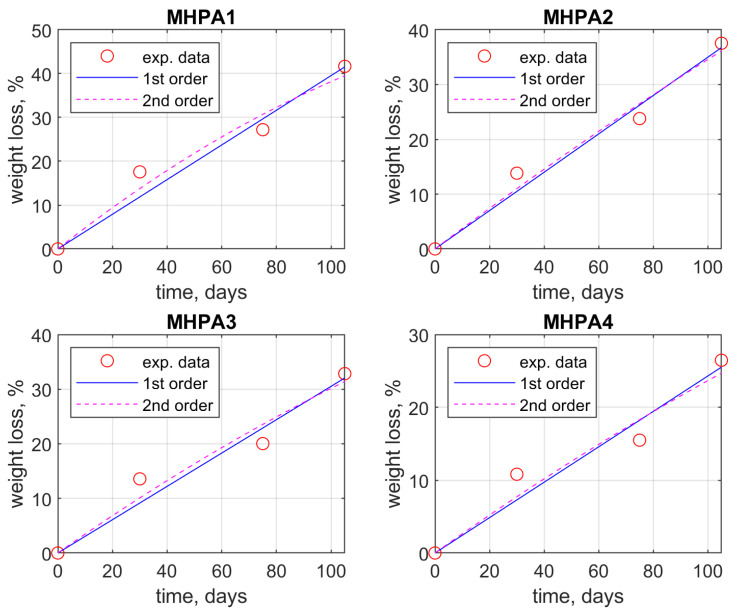
Second-order polynomial fitting regression model (blue line) and linear fit (black line) applied against the weight loss profile (red dots) of M_HPAx (x = 1, 2, 3, 4) glycopolymer samples during the soil biodegradation process.

The data concerning the weight loss of the glycopolymers at given times (30, 75, and 105 days) during the biodegradation process in soil were analyzed using Matlab software. From a kinetic point of view, the rate of the biodegradation process can be illustrated by expressing the weight loss dependency in time. Figure 4 presents the regression curves obtained by applying polynomial fitting to data using the *polyfit* and *polyval* modeling functions available for linear and second-order polynomials, respectively. For accurate fitting of the data, Matlab modeling was performed by imposing the restriction that the linear and polynomial curves should pass through the origin of the coordinate system, as from a kinetics point of view, at time zero there is no registered weight loss.

The correlation and root mean standard coefficients were calculated for each mathematical model in order to ensure a reasonable fit of the data. Generally, it is accepted that the correlation coefficient should be as close to unity as possible for the mathematical model to reflect the behavior of the system, while the root mean standard deviation value is recommended to be as low as possible (close to zero) [46].

Table 2 presents the mathematical equations concerning the correlation between weight loss and time in the form of linear dependency and second-degree polynomials, respectively, and the corresponding correlation coefficient and square-mean correlation deviation as an adequacy assessment of the employed model. As discussed beforehand, the intercept of the equations is equal to zero as a limitation imposed to fit the biodegradation of the samples. It is noteworthy that the R^2^ coefficient is satisfactory for linear dependency; however, for the polynomial fit, it is somewhat better. Nonetheless, the root-mean standard deviation (RMSD) has smaller values for the linear models, reflecting the dependency of weight loss on time in all cases registered.

**Figure 5:**
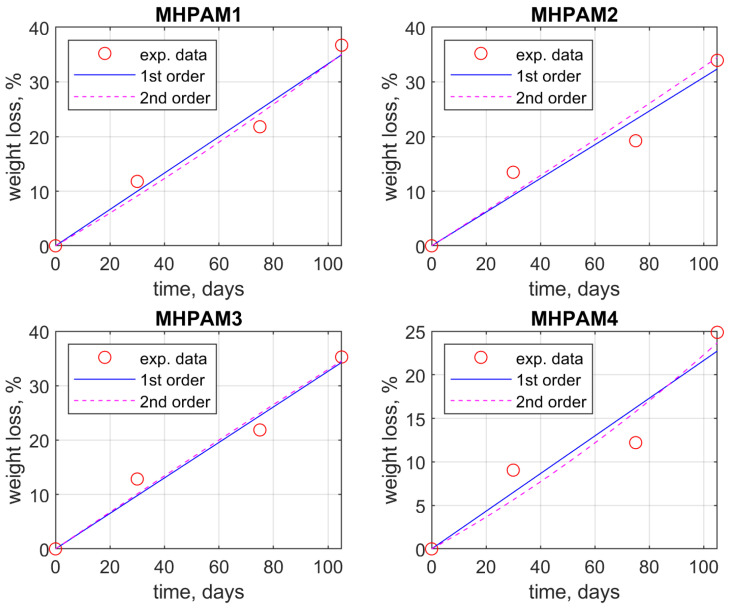
Second-order polynomial fitting regression model (blue line) and linear fit (black line) applied against the weight loss profile (red dots) of M_HPMAx (x = 1, 2, 3, 4) glycopolymer samples during the soil biodegradation process.

The same approach was employed in the case of data from biodegradation in soil performed on glycopolymers derived from D-mannose oligomer and hydroxypropyl methacrylate. The weight loss was fitted against time using a linear and a second-degree polynomial equation using the Matlab polyfit function. Figure 5 displays the experimental data and the mathematical models for the M_HPMAx samples.

Matlab software enabled the calculus of linear and polynomial coefficients, as well as the correlation coefficients, which proved a good fit for the linear model for the weight loss profile (Table 3). As a limitation imposed on the mathematical models developed using Matlab, the linear and polynomial dependency is required to pass the origin in all analyzed cases. 

Even though the correlation coefficients R^2^ display better values for the 2nd-degree polynomials that would recommend them as mathematical models to describe the biodegradation process, the root-mean standard deviation (RMSD) value is merely higher for the polynomial fit, thus the linear dependency would be adequate enough to characterize the process.

According to the mathematical models, it appears that the glycopolymers could lose most of their weight in about a year, especially the ones containing a higher sugar content. However, this assumption would only be accurate if the biodegradation tendency in terms of kinetics remained the same during the whole process. It is possible, though, for the biodegradation process to reach a plateau stage, corresponding to a saturation phase, which is often encountered in biotechnological processes dependent on microorganisms’ development. Sadly, the assessment of weight loss for the biodegradation process could not be carried out for a longer period of time due to the fact that the samples became too frail to dig out in full, and further investigation would have been inexact. 

The mathematical modeling of the biodegradation process during the burial of the glycopolymer samples in soil could have been more accurate if more data were provided by washing the fragments more frequently. However, this procedure was considered to hinder the biodegradation process since the microorganisms that developed on the surface of the sample were removed by washing every time the weighing procedure was carried out. 

Also, if the experiment was continued after the 105 days, valuable data could be found concerning the biodegradation pattern of the glycopolymer samples discussed herein.

## 4. Conclusions

Glycopolymer plastic materials derived from D-mannose with acrylate and methacrylate moieties present good biodegradation features after 14 days of incubation in liquid media containing *Proteus mirabilis*, losing almost 50% of their weight and showing alteration of their surface, a fact proved by SEM imaging. Also, the C:O ratio is modified after the *P. mirabilis* action, which could explain the use of a certain amount of polymeric chain as a carbon source for microorganisms’ proliferation.

The polymeric samples derived from the same D-mannose oligomer and the acrylic and methacrylic monomers in four different weight ratios were tested for biodegradation by burial in garden soil for about 100 days. The polymer derived from D-mannose oligomer and acrylate containing the most sugar moiety displayed the best biodegradation profile, losing more than 40% of its weight in 105 days, while the others lost more than 25% of their weight in all cases discussed. The biodegradation pattern was fitted against linear and polynomial mathematical models using Matlab software. It must be mentioned that a linear dependency between weight loss and time would be adequate to describe the biodegradation process in soil; nonetheless, the calculated correlation coefficients (R^2^) for the 2nd degree polynomial are superior.

According to the models, the biodegradation of the samples could be achieved in about a year for most of the tested samples.

## Figures and Tables

**Table 1:** Weight loss of glycopolymer samples after biodegradation in soil.

	Sample	Weight Loss, %			
Time, Days		0	30	75	105
M_HPA1		0	17.57	27.2	41.65
M_HPA2		0	13.82	23.79	37.54
M_HPA3		0	13.58	20.04	32.84
M_HPA4		0	10.82	15.5	26.47
M_HPMA1		0	11.79	21.79	36.72
M_HPMA2		0	13.46	19.23	33.95
M_HPMA3		0	12.85	21.87	35.26
M_HPMA4		0	9.05	12.21	24.87

**Table 2:** Mathematical models of M_HPAx (x = 1, 2, 3, 4) glycopolymers biodegradation in soil.

Sample	Mathematical Model	Root-Mean Standard Deviation (RMSD)	Correlation Coefficient (R^2^)
M_HPA1	w = 0.3955 · t w = −0.011 · t^2^ + 0.4917 · t	3.5882 3.9334	0.9578 0.9662
M_HPA2	w = 0.3499 · t w = −0.0003 · t^2^ + 0.3757 · t	2.4288 2.9278	0.9765 0.9772
M_HPA3	w = 0.3053 · t w = −0.0005 · t^2^ + 0.3516 · t	3.0727 3.6435	0.9494 0.9526
M_HPA4	w = 0.2431 · t w = −0.0003 · t^2^ + 0.2667 · t	2.6330 3.1889	0.9424 0.9437

where: w—weight loss, %, t—time, days.

**Table 3:** Mathematical models of M_HPMAx (x = 1, 2, 3, 4) glycopolymers biodegradation in soil.

Sample	Mathematical Model	Root-Mean Standard Deviation (RMSD)	Correlation Coefficient (R^2^)
M_HPMA1	w = 0.3330 · t w = 0.0004 · t^2^ + 0.2920 · t	2.3427 2.7453	0.9773 0.9793
M_HPMA2	w = 0.3083 · t w = 0.0001 · t^2^ + 0.3177 · t	3.4339 4.2013	0.9404 0.9405
M_HPMA3	w = 0.3264 · t w = −0.0001 · t^2^ + 0.3397 · t	2.3902 2.9148	0.9741 0.9743
M_HPMA4	w = 0.2164 · t w = 0.0005 · t^2^ + 0.1729 · t	3.0176 3.5881	0.9140 0.9189

where: w—weight loss, %, t—time, days.

## Data Availability

The data presented in this study are available on request from the corresponding author. The data are not publicly available due to further studies possible leading to a patent proposal.
